# Artificial intelligence planning and 3D printing augmented modules in the treatment of a complicated hip joint revision: a case report

**DOI:** 10.3389/fsurg.2023.1237075

**Published:** 2023-09-19

**Authors:** Yikai Liu, Zian Zhang, Wenzhe Wang, Chaoqun Yu, Chang Liu, Zhenchao Huang, Kaige Xu, Haining Zhang

**Affiliations:** Department of Joint Surgery, The Affiliated Hospital of Qingdao University, Qingdao, China

**Keywords:** three-dimensional printing, artificial intelligence planning, total hip revision, osseous defectdefects, total hip arthroplasty

## Abstract

Total hip revision with osseous defects can be very difficult. Artificial intelligence offers preoperative planning, real-time measurement, and intraoperative judgment, which can guide prothesis placement more accurately. Three-dimensional printed metel augment modules which are made according to the individualized osseous anatomy, can fit the osseous defects well and provide mechanical support. In this case, we used AI to plan the size and position of the acetabular cup and 3D-printed augmented modules in a complicated hip revision with an acetabular bone defects, which achieved stable fixation and relieved hip pain postoperatively.

## 1. Introduction

Three-dimensional (3D) printing can be used to create a specific structure and has achieved great success in facilitating surgical procedures or producing structural support that fits the defects of the disease region (1). In medicine, 3D printing is based on computed tomography (CT) or magnetic resonance imaging data. A duplicate of the bone structure can be obtained by 3D printing, which allows orthopedists to rehearse complicated surgeries before operating on patients (2). In addition, when facing osseous defects, especially large defects, the autogenous bone may not satisfy the requirement, and allogenic bone granules can be extremely expensive; however, 3D printing modules can fit the bone defects well with rare complications (3). Attempts to use 3D printing to model complex pelvis and acetabulum fractures have been successful (4). Moreover, a 3D print-prototyping pelvic model was reported to facilitate preoperational planning of developmental dysplasia in patients undergoing total hip arthroplasty (THA) (5).

Recent evidence has confirmed the efficacy of artificial intelligence (AI) in orthopedic surgeries (6), due to its advantages in preoperative planning, real-time measurement, and intraoperative judgment. Therefore, AI has the potential to revolutionize surgery and optimize the quality of patient care in the future (7). In this case, we used AI to plan the size and position of the acetabular cup and 3D-printed augmented modules in a complicated hip revision with an acetabular bone defects, which achieved stable fixation and relieved hip pain postoperatively.

## 2. Case presentation

A 66-year-old woman with a history of THA for 30 years was admitted to our hospital with “right hip pain.” Thirty years ago, the patient experienced right hip pain, was diagnosed with “avascular necrosis of the femoral head,” and underwent THA. Pain in the right hip was relieved postoperatively. Thirteen years ago, the hip joint pain aggravated without apparent reason, and she was diagnosed with “liner abrasion,” and a revision surgery of the total hip was performed. The pain in the right hip was well-controlled after revision. Three years ago, the pain in the right hip aggravated again without obvious precipitating factors and could not be controlled by nonsurgical methods, and the visual analogue scale (VAS) was 7; therefore, she visited our hospital for surgical treatment.

**Figure 1:**
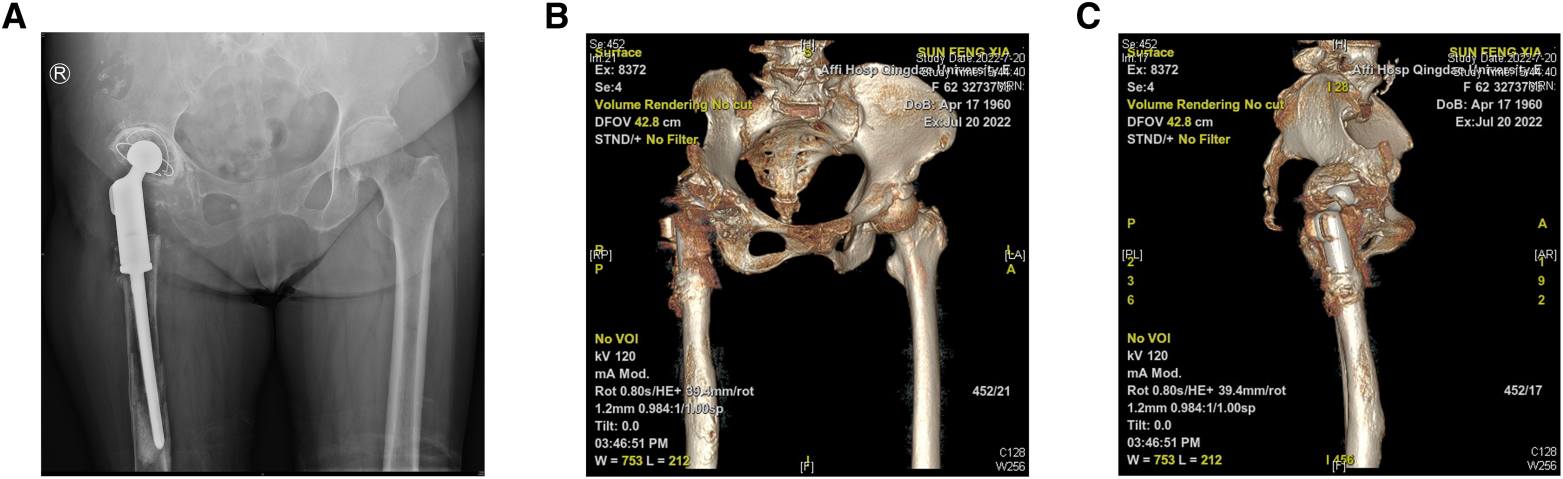
(**A**) A pelvic radiograph (anteroposterior view) shows the right acetabular prosthesis moving up significantly and the right femoral prosthesis sinking, with a slight and massive osseous defects on the pubis and proximal femur, respectively. (**B,C**) Three-dimensional computed tomography reconstruction reveals the abnormal position of the prosthesis and osseous defects.

**Figure 2:**
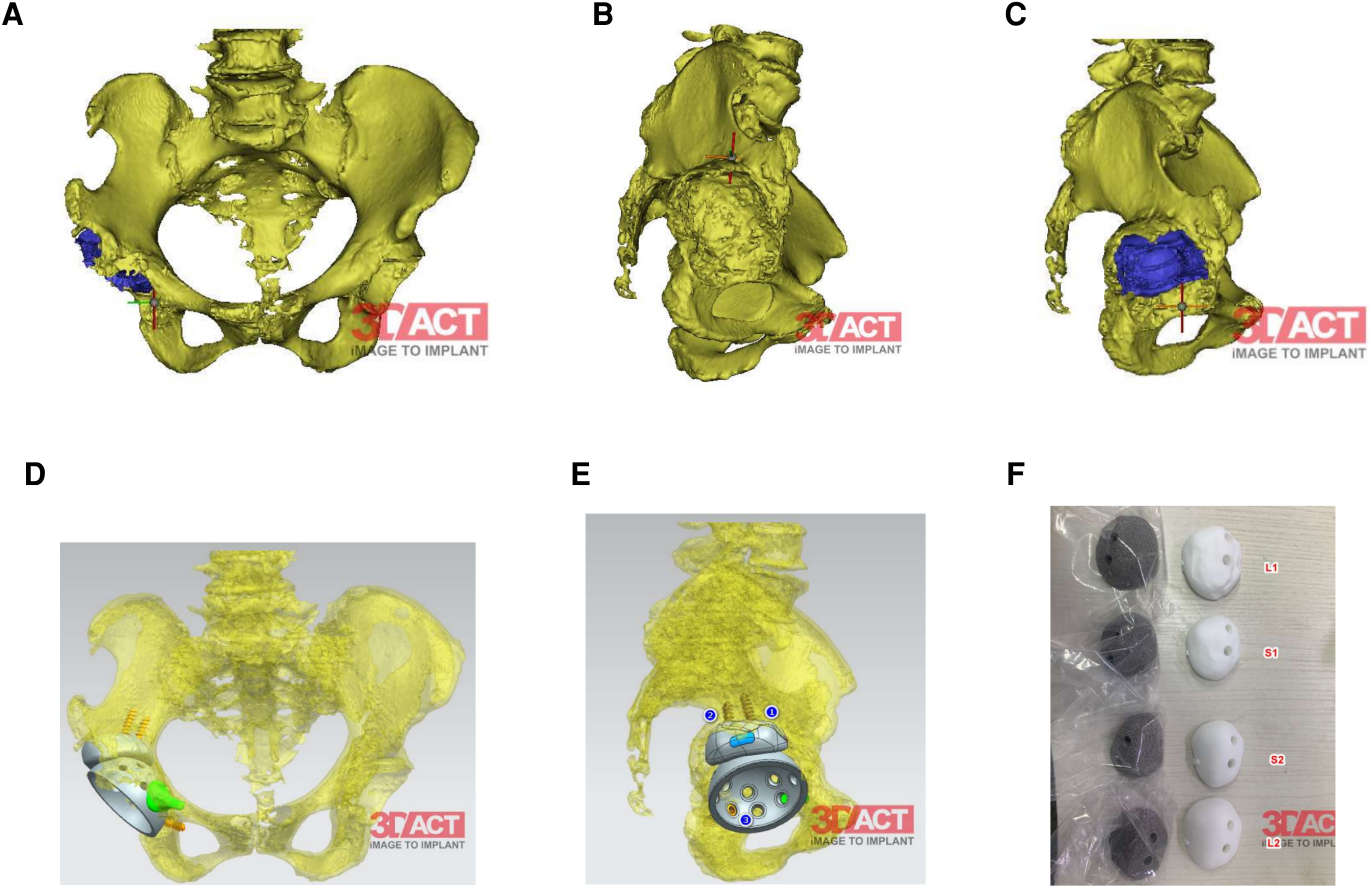
(**A,B**) Right acetabulum bone defects shown by 3D-reconstruction. (**C**) The preoperative position planning of the cup and the rotational center of hip. (**D,E**) The preoperative position planning of the cup, augments and the arrangement of the screws. (**F**) Four different test models and prothesis varing in size and the surface (smmoth or rough). The size and the surface depend on the condition of the bone bed after removing the residual bone cement.

The preoperative imaging data of the patient showed that the right acetabular prosthesis had moved up significantly, and the right femoral prosthesis had sunk (Figure 1). Thus, we decided to perform a total hip prosthesis revision surgery. The AI planning and 3D printing was conducted by AKMEDICAL company. 3D CT data of the patients were collected preoperatively, and computer-aided design (CAD) software was used to design the size and position of implants based on the specific needs of patients and the advice of doctors. Then the designed implant model was converted into STL (Standard Tessellation Language) format that can be used for 3D printing, which was performed using EBM Q10Plus (Arcam, Sweden). Several digital images of the preoperative planning are shown in Figure 2, and the detailed design is attached in the Supplementary file 1. The patient's right acetabulum moved upward. We planned to place the reinforcement blocks at the bone defects to provide sufficient coverage and support to the acetabulum. Augments were made by 3D printing technology to match the patient's anatomical structure.

**Figure 3:**
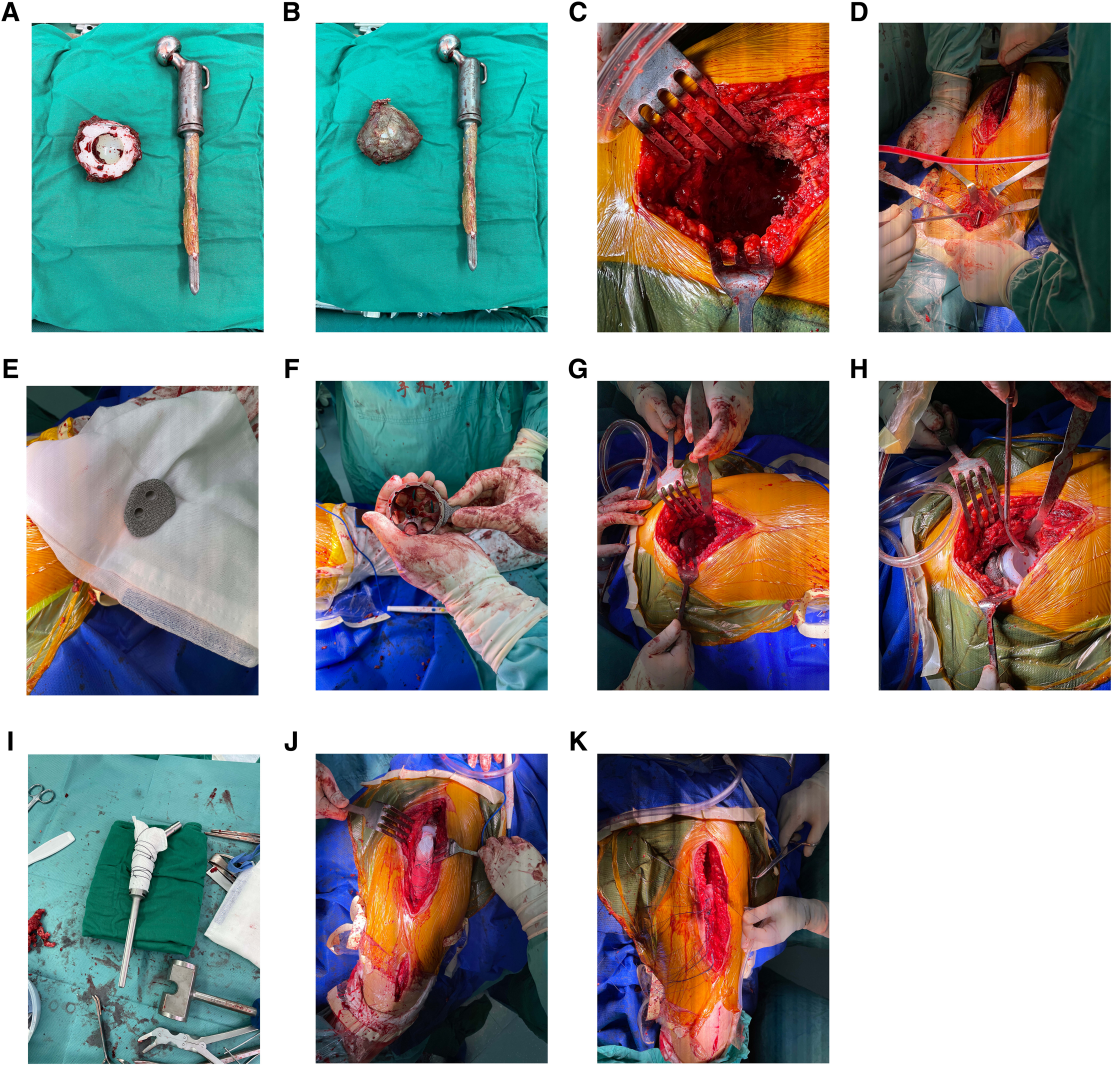
(**A,B**) Acetabular and femoral prostheses were removed intraoperatively. Residual bone cement was observed on the surface of the femoral prosthesis. (**C**) The appearance of the acetabulum after taking out the acetabular prosthesis. (**D**) A bone window with a length and width of approximately 1.3 cm–4 cm was created at the lateral side of the distal femur to remove the residual bone cement at the distal femoral medullary cavity and establish the true bone canal with a bone graft at the distal femur. (**E**) The artificial intelligence-designed three-dimensional-printing porous metal augmented modules were used to fill the bone defects at the external superior of the acetabulum to provide sufficient mechanical support for the revision acetabular prosthesis. (**F**) The artificial intelligence-designed positions of the augmentation modules were used to fill the pubic bone defects. (**G**) An augmented module was placed at the external superior of the acetabulum. (**H**) The placement of two augment modules, an acetabular cup, and a cup liner. (**I**) The proximal end of the femoral prosthesis wrapped the Lars ligament. (**J,K**) The muscles were sutured and fixed on the Lars ligament.

**Figure 4:**
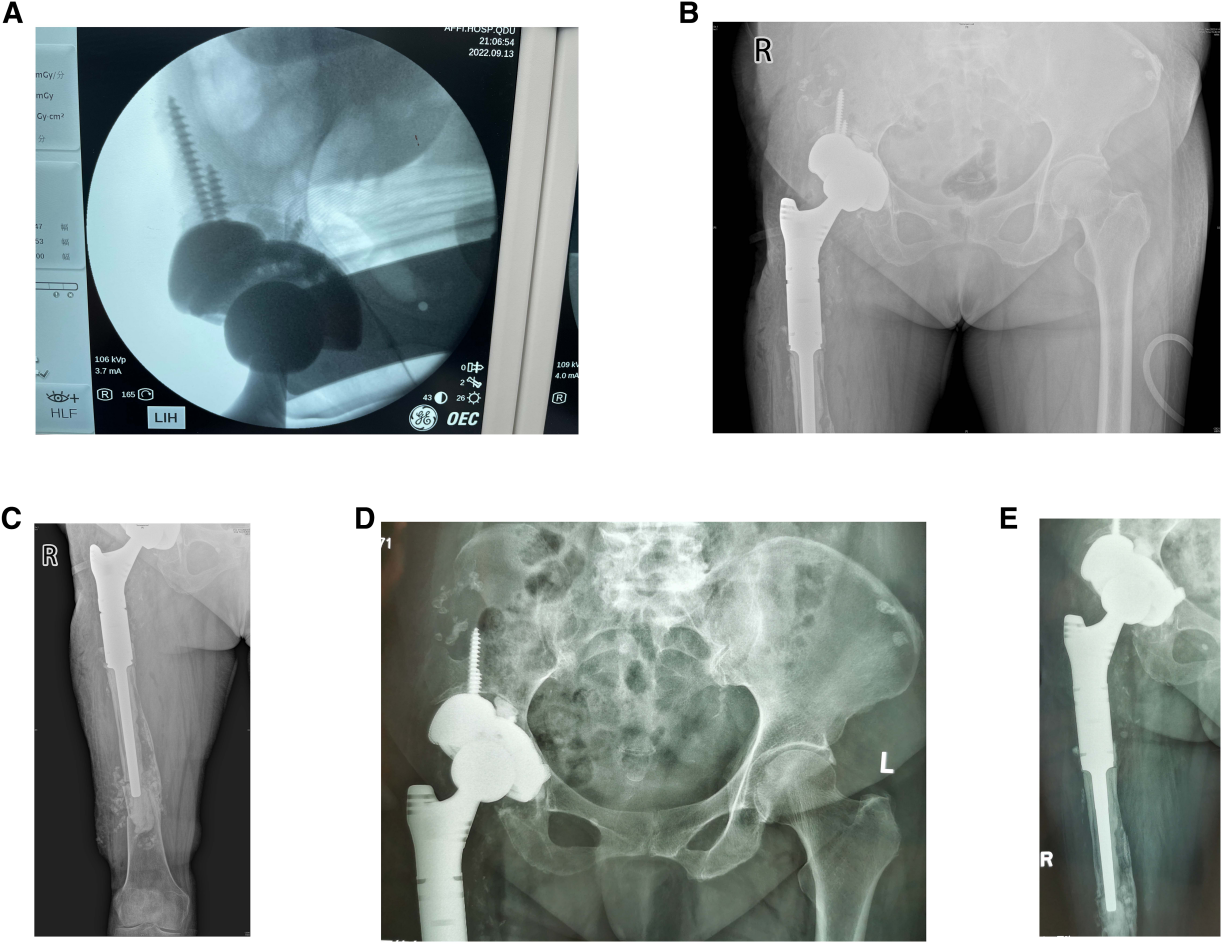
(**A**) Intraoperative C-arm radiograph showed a good position of augmented modules and prostheses. (**B,C**) Radiographs of the hip and femur were taken at the bedside 1 day postoperatively. (**D,E**) Radiographs of the hip and femur were taken at the outpatient clinic 5 weeks postoperatively.

Revision surgery was performed under general anesthesia. The hip joint was exposed using the modified Hardinge's approach. After separating the subcutaneous tissue and fascia lata, severe scar tissue and adhesions were observed in the soft tissue, and the anatomical structure was unclear. The surgical scars and abnormal synovium were removed to expose the femur. A loose femoral prosthesis was also observed. We removed the femoral prosthesis and cleaned the surrounding necrotic and pseudotumor tissues (Figures 3A,B). After exposing the acetabulum, we observed a large amount of bone absorption around the acetabular prosthesis (Figure 3C). The acetabular prosthesis was fixed using bone cement. We removed the acetabular prosthesis and reamed the acetabulum with a 60 mm acetabulum reamer (Figures 3A,B). We drilled and created a bone canal for screws and placed a 60 mm acetabular prosthesis and two augments that filled the osseous defects (Figures 3E–G). The acetabular prosthesis was fixed with bone cement, considering the complicated bone bed of the acetabulum. A 36 mm acetabulum liner was selected (Figure 3H). We then used a long osteotome and grinding drill to clean the residual bone cement in the distal femur. A window was opened at the lateral side of the distal femur, with a length and width of approximately 1.3 cm × 4 cm, to remove the residual bone cement at the distal end and establish the true bone canal with a bone graft at the distal femur (Figure 3D). After placing the distal plug of the prosthesis and the bone cement injection, a 120 mm femoral prosthesis was placed immediately, followed by replanting the removed bone flap in the distal femur. Subsequently, a 36S ceramic femoral head prosthesis was placed. The hip was stable after the femoral head reduction. After irrigating the joint with a large amount of normal saline, 1 g of vancomycin powder was sprinkled into the joint cavity to prevent infection. The proximal end of the femoral prosthesis wrapped the ligament augmentation and reconstruction system (LARS) ligament, and the muscles were sutured and fixed (Figures 3I–K). A C-arm radiograph showed good positioning of the prosthesis and augmented modules (Figure 4A). We closed the incision layer-by-layer and ended the procedure. The patient underwent rehabilitation training postoperatively and was discharged 2 weeks later.

On the first day after surgery, bedside radiography showed good positioning of the prosthesis and augmented modules (Figures 4B,C). The pain was markedly relieved 1 week postoperatively, and the VAS was 3. The prosthesis and augmented modules were still fixed well to the surrounding bone bed, and no prosthesis loosening was observed at the 5-week follow-up (Figures 4D,E) and the VAS was 1, which indicated a good short-term outcome. The patient was satisfied with the revision surgery.

## 3. Discussion

AI preoperative planning and 3D-printing has been shown effective in restoring rotation center in total hip arthroplasty for the treatment of developmental dysplasia of the hip (8). However, the use of this technique in total revision surgery with large bone defects is relatively rare. We used AI preoperative planning and 3D-printing porous metal augmentation modules to reconstruct the acetabular position accurately. Although an ideal acetabular position can be obtained using imaging data preoperatively, it is difficult to reconstruct the acetabulum intraoperatively accurately. Preoperative planning is indispensable in determining the acetabular reaming depth and cup size. The design of 3D printing augments modules can provide better support for the acetabulum and ensure the initial stability of the hip joint. The matching degree of augmentation between the bone bed and acetabulum is crucial for hip joint stability postoperatively. If there is a gap between the prosthesis and the bone bed, or if the augmentation position is poorly placed, it will be difficult to achieve early stability and long-term bone growth (9). Therefore,it is essential to use augment modules and to place the prothesis accurately.

Another advantage of the AI design is that we can use a test model for rehearsal before placing the acetabular prosthesis; the test models were also made by 3D printing according to preoperative images. Prefabricated screw holes were present on the acetabular and augmented test models, which allowed surgeons to drill holes and mark the position of the augmentation and acetabular prostheses.

The patient used a bone-cement prosthesis for the last revision. Therefore, when we removed the femoral prosthesis, some residual bone cement in the medullary cavity of the distal femur was difficult to remove. We fenestrated the distal femur to completely remove the bone cement adhering to the inner wall of the medullary cavity and re-establish the normal prosthesis canal. Cementless, extensively porous-coated stems can bypass the proximal femoral bone defects region and achieve reliable fixation depending on 5–7 cm of the diaphysis and have produced reliable clinical and radiographic results in revision THA with femoral bone loss (10). However, extensively porous-coated stem application in femurs with severe osseous defects remains a concern because the bone defects involved the diaphysis in our case, and the residual diaphyseal bone may be inadequate for distal fixation (9, 11). Moreover, the sinking of the femoral side prosthesis led to significant changes in the shape of the femoral medullary cavity, probably with a small amount of bone cement remaining in the inner wall of the femur, which was difficult to remove completely. For these reasons, we perceived that the biotype-designed prosthesis might have difficulty achieving stable fixation; therefore, we still used bone-cement fixation in this revision surgery. For the removal of the residual bone cement, one study suggested that a flexible endoscope can be applied to the cemented femoral medullary canal to obtain better visual field, confirm the status of the bone bed and assist surgical procedures in total hip revision arthroplasty (12).

Finally, part of the hypertrophic scar and abnormal soft tissue was removed intraoperatively, which made it challenging to sew the muscle together. Therefore, the LARS artificial ligament was used to wrap the femoral prosthesis before placing it in the medullary cavity and fixing the tensor fascia lata to the ligament.

There are other approaches using for the treatment of bone defects during hip revision. Traditionally, structural bone graft is being used to treat acetabular superior lateral wall defect, and its effect depends on the size, orientation, and method of fixation of the allografts as well as adequate remaining host-bone (13). There are also some different types of acetabular reinforcement rings are used to reconstruct the acetabular bone defects, however, it has drawbacks, for example, potential impingement of the sciatic nerve caused by mal-positioning and the intra-operative re-shaping of the implant by the surgeon (14). By comparision, AI preoperative planning can largely reduce the occurrence of this problem. One study reported a satisfactory initial stability in hip revision using a cementless, dual mobility implant with a peg on the cup. The average length of the peg is 69 mm, with a range of 55–80 mm, and primary fixation in healthy bone is performed by anchoring the peg in the iliopubic beam (15).One meta-analysis suggests that cup-cage construct can reach a good clinical outcome with a low complication and revision rate, which is a promising method for treating huge acetabular bone defects in total hip revision (16). Moreover, one study designed an implant which provides optimal electric fields in the acetabular region to accelerate the healing of bone defects, enhance the reconstruction of the pelvic bone and improve the fixation of the prosthesis (17). Another study proposed a novel technique: the bone defects were reconstructed through the Stoppa approach combined with the lateral window of ilioinguinal approach by means of bone struts and metallic plates, followed by reconstructing the acetabulum using porous tantalum augments and morselized allograft in an extended posterolateral hip approach, then a cemented constrained socket was implanted. The patient was able to walk with one crutch without pain at the one-year follow-up, which suggested a satisfactory clinical outcome of this technique (18).

## 4. Conclusion

In this case report, we tested the efficacy of AI preoperative planning and 3D-printing metal augmentation modules in treating hip revision with huge acetabular and proximal femur osseus defects. The accurate placement of prothesis and use of augmentation module reached good short-term clinical outcome.

## Data Availability

The original contributions presented in the study are included in the article/Supplementary Material, further inquiries can be directed to the corresponding author.
