# Supplementary material for: Artificial intelligence planning and 3D printing augmented modules in the treatment of a complicated hip joint revision: a case report
Source: Front Surg. 2023 Sep 19;10:1237075. doi: 10.3389/fsurg.2023.1237075 (PMC10546305; doi:10.3389/fsurg.2023.1237075)
Supplement: Supplementary file 1 [file Image1.pdf]

手术规划

项目编号： 3DB221303

患者编号： 20203130303-0083

患者姓名：**Sun Fengxia**

主任医师：张海宁

医院：青岛大学附属医院

规划包含:

- 假体摆放模拟
- 钉道方向及长度

手术规划:王好奎
假体设计:王好奎

一、病例信息

- 病人：女，62
- 病情信息：右侧髋臼缺损
- 数据扫描日期：未知
- 数据接收日期：2022/7/29
- 手术日期：待定

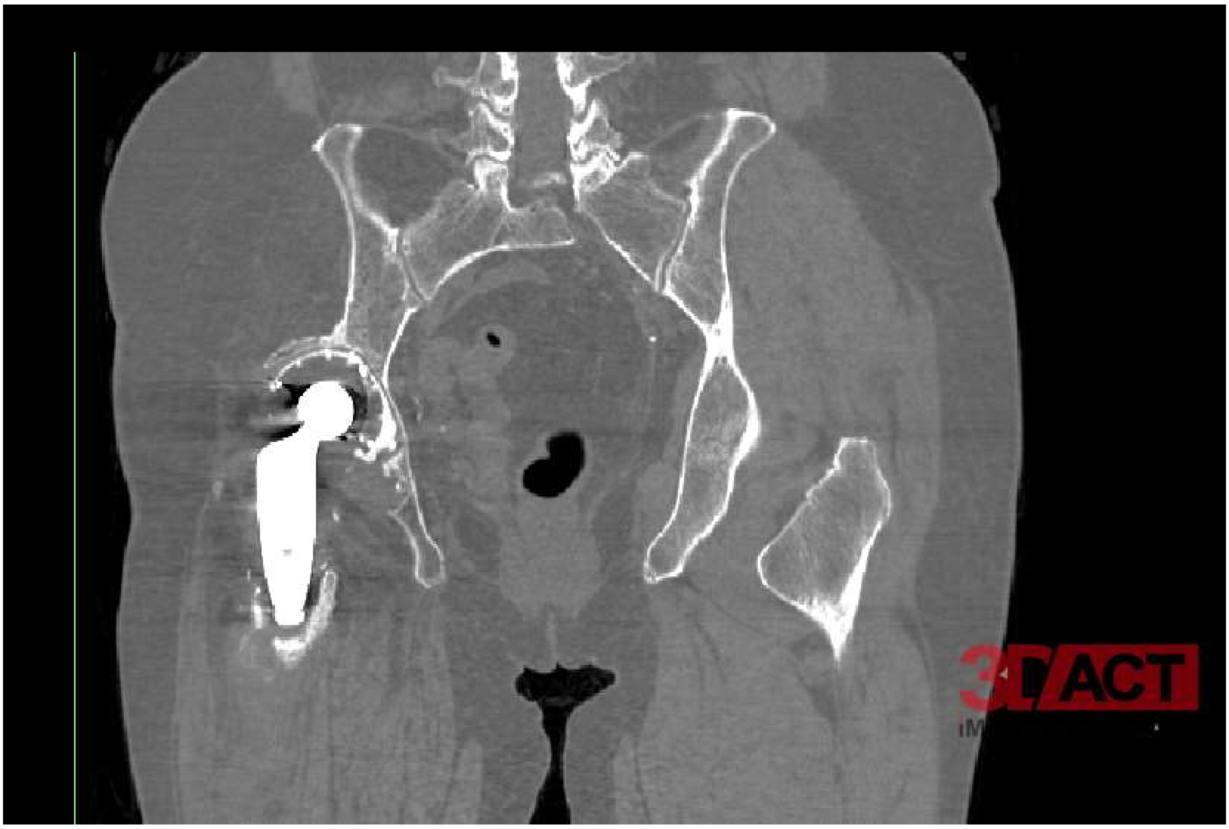

二、术前说明

右侧髋臼部分，由于骨水泥的存在，使得髋臼重建存在偏差

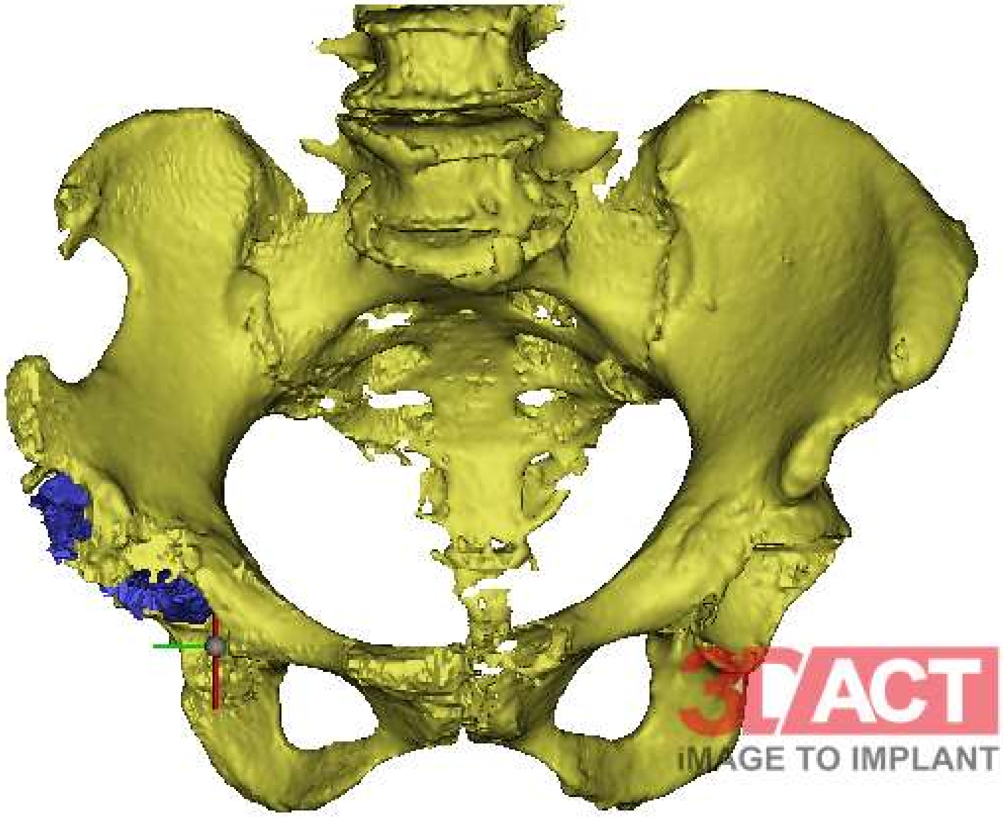

右侧髋臼预览

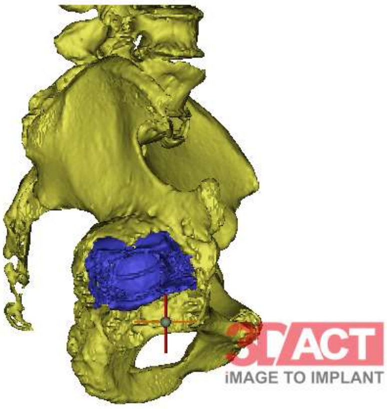

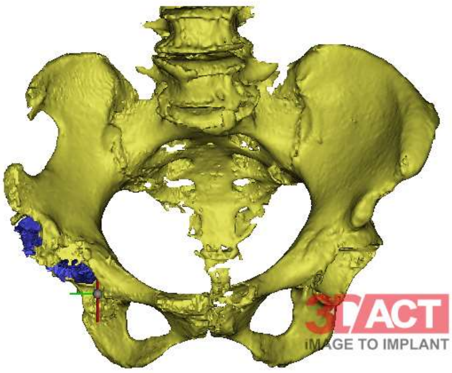

水泥去除后。右侧髋臼重建

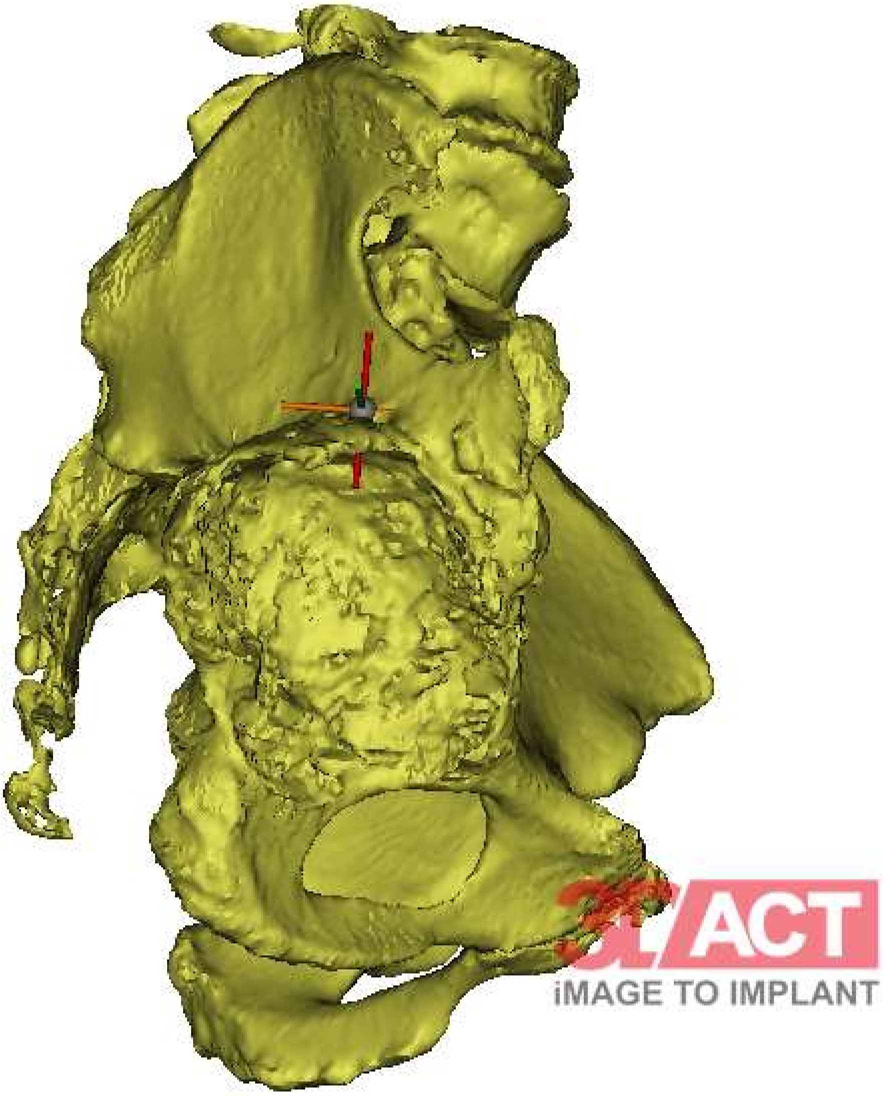

三、假体设计说明

髋臼杯摆放说明
前倾角：15°
外展角：45°
杯大小
外径：64mm

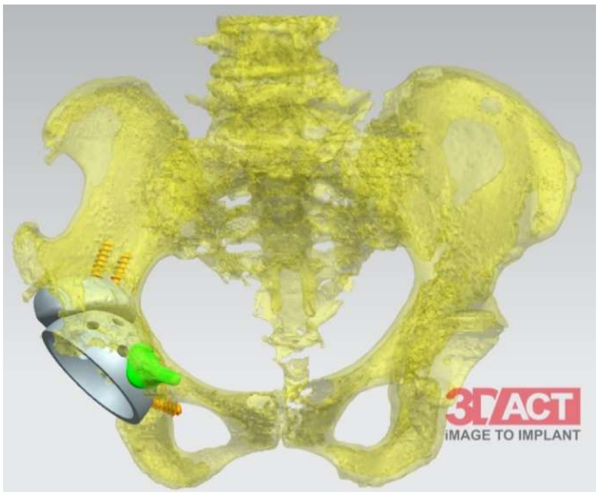

冠状位透视图

四、假体设计方案

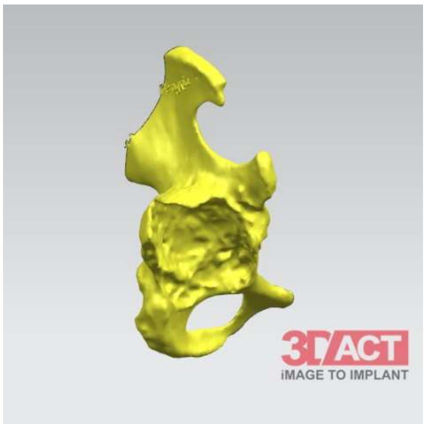

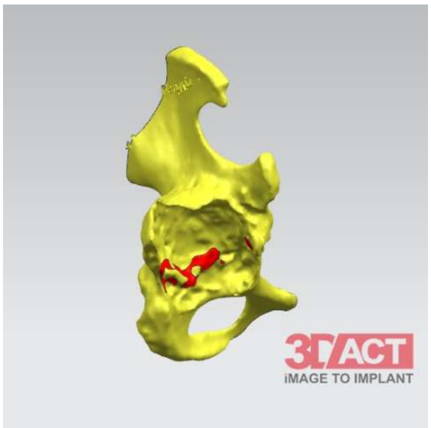

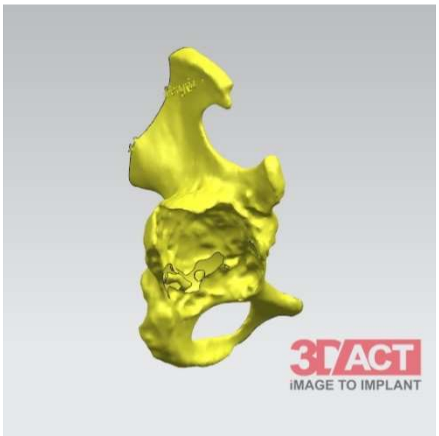

左侧髋臼重建　　红色为去除骨质　　去除骨质后形态

五、螺钉长度说明

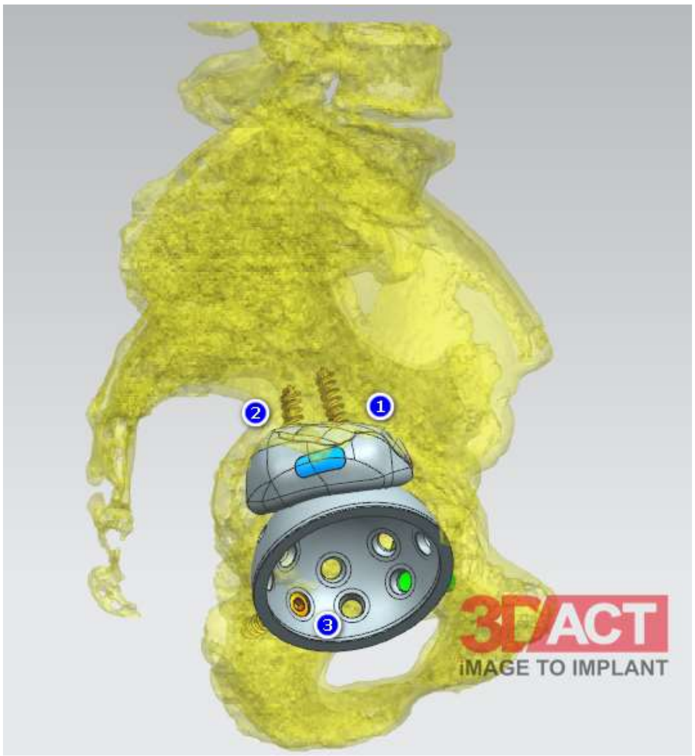

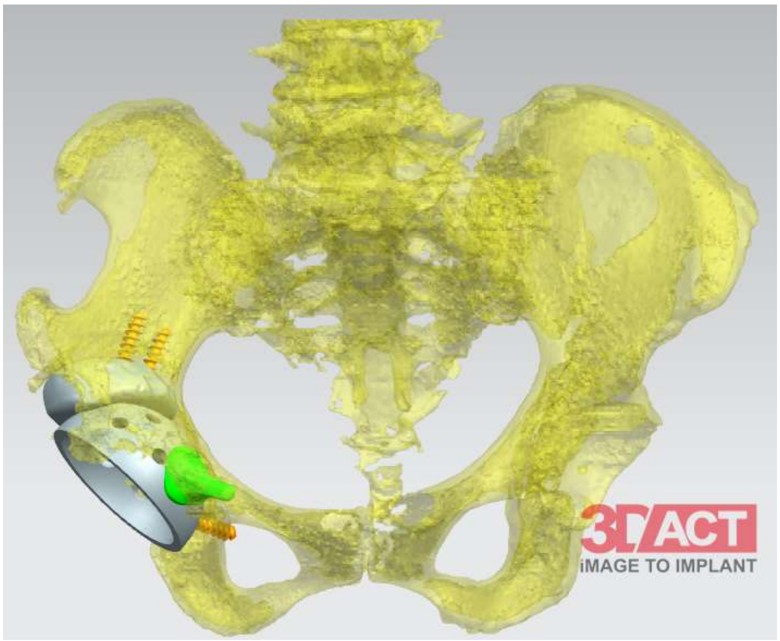

<table>
<tr><th>螺钉编号</th><th>螺钉长度（mm）</th><th>螺钉直径（mm）</th></tr>
<tr><td>1</td><td>75</td><td rowspan="3">6.5</td></tr>
<tr><td>2</td><td>94</td></tr>
<tr><td>3</td><td>16</td></tr>
</table>

注：表中标注螺钉长度为图中模拟钉道方向螺钉未穿出骨质时最大长度。

六、假体规格说明

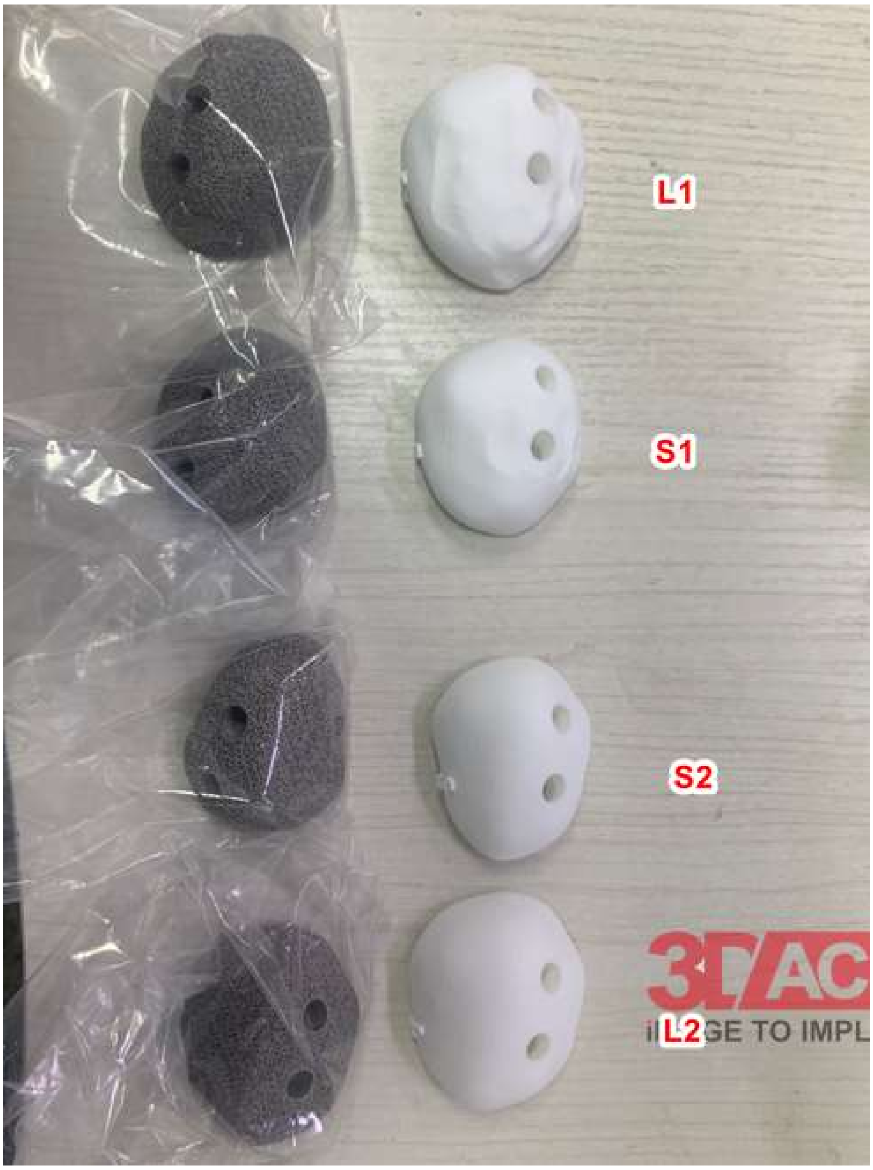

本次假体打印共计 **4** 个规格，分别为 **L1**、**S1**、**L2**、**S2**，其区别如下表所示：

假体规格	区别
**L1 S1**	贴合骨面设计，设置了大小号，小号整体比大号小 **2mm**，术中可根据骨质情况进行选择。
**L2 S2**	骨面贴合处，光滑设计，设置了大小号，小号整体比大号小 **2mm**，术中可根据骨质情况进行选择。
